# The Earth Closet System

**Published:** 1871-05

**Authors:** 


### The Earth Closet System.

We had occasion, says the *Journal of Applied Chemistry*, during the summer, to subject the earth closet system to a thorough test, and are so fully convinced of its practicability and efficacy that we deem it our duty to publish the results of our experiments. We prepared the earth by passing it through a sieve such as masons use, and allowing it to dry in the sun. A few minutes sufficed to get ready enough to last a week, and, as we used the same earth over again five or six times, the labor and trouble of this part of the operation was very stight. We had a self acting seat, and a hopper large enough to hold dry earth sufficient for a week's supply for a family of five persons. The tank under the seat was made of wood. on runners, so that it could easily be run out into a wheel-barrow, ready for dumping. A more convenient method would have been to put this on wheels, ready to remove to the shelter for drying. When the tank was full it was emptied upon a floor under an open shed, and in a day or two the earth was usually sufficiently dry to be employed again. after the earth had been used five times it had the odor of guano, but was not in the least offensive. There was not the least smell observable in the closet, so that we had it constructed under our piazza, and could have used our commode in any apartment of the house without the slightest inconvenience.

So far as disagreeable smell is concerned, we did not fully appreciate the great advantages of the earth system over the water closets until we came to town, and we should be glad to be able to use earth in the city on the score of its freedom from the unhealthy smell that attends the employment of water if it could be obtained from dealers, and could be called for by the drivers of ash carts. We have no doubt that, in the course of time, dry earth will be substituted for water in a majority of our best city houses. The open privy system of the country is the occasion of much sickness, and is such an unmitigated nuisance, that it ought to be prohibited by law. It poisons the air, fills the well water with organic matter, and produces fevers and cholera. There is really no valid excuse for not introducing the earth closet system in the country, and we are of the opinion that nothing but ignorance stands in the way of its universal adoption.

